# Supplementary material for: Detection of SARS-CoV-2 RNA using RT-LAMP and molecular beacons
Source: Genome Biol. 2021 Jun 3;22:169. doi: 10.1186/s13059-021-02387-y (PMC8173101; doi:10.1186/s13059-021-02387-y)
Supplement: Supplementary file 1 — Additional file 1: Figures S1-S3. [file 13059_2021_2387_MOESM1_ESM.pdf]

Figure S1

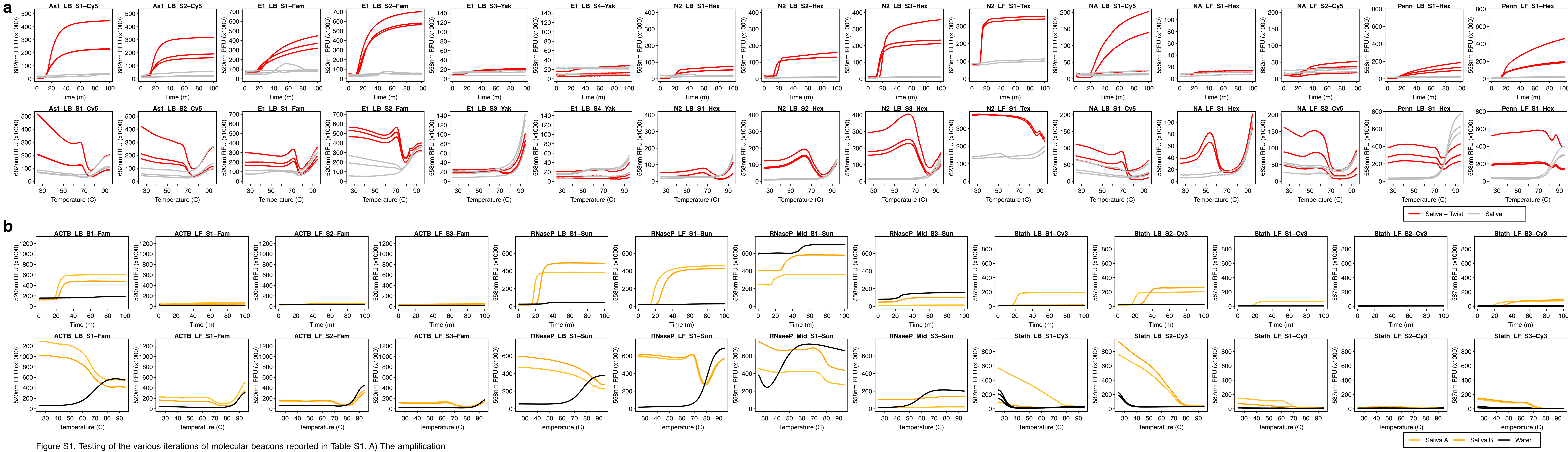

Figure S1. Testing of the various iterations of molecular beacons reported in Table S1. A) The amplification and melt curves of various SARS-CoV-2 targeted beacons. Each plot shows amplification and melt curves in reactions amplifying saliva (grey) or saliva doped with 10,000 copies of Twist synthetic SARS-CoV-2 RNA (red). Beacon and fluorophore identity is indicated above each plot. B) As in A but for beacons targeting human sequences. Amplification was performed on saliva from two different individuals (yellow or orange) or water (black).

Figure S2

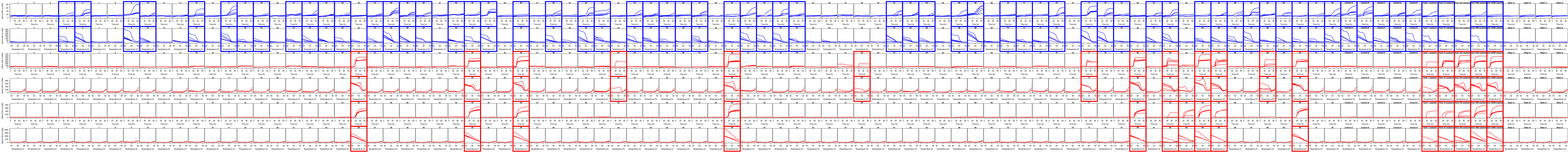

Figure S2. Reaction progression and melt curves for LAMP-BEAC reactions carried out on clinical saliva samples. Each column represents a single sample assayed. Sample names are as in Table S2. Heavy boxes indicate samples called positive for that amplicon.

Figure S3

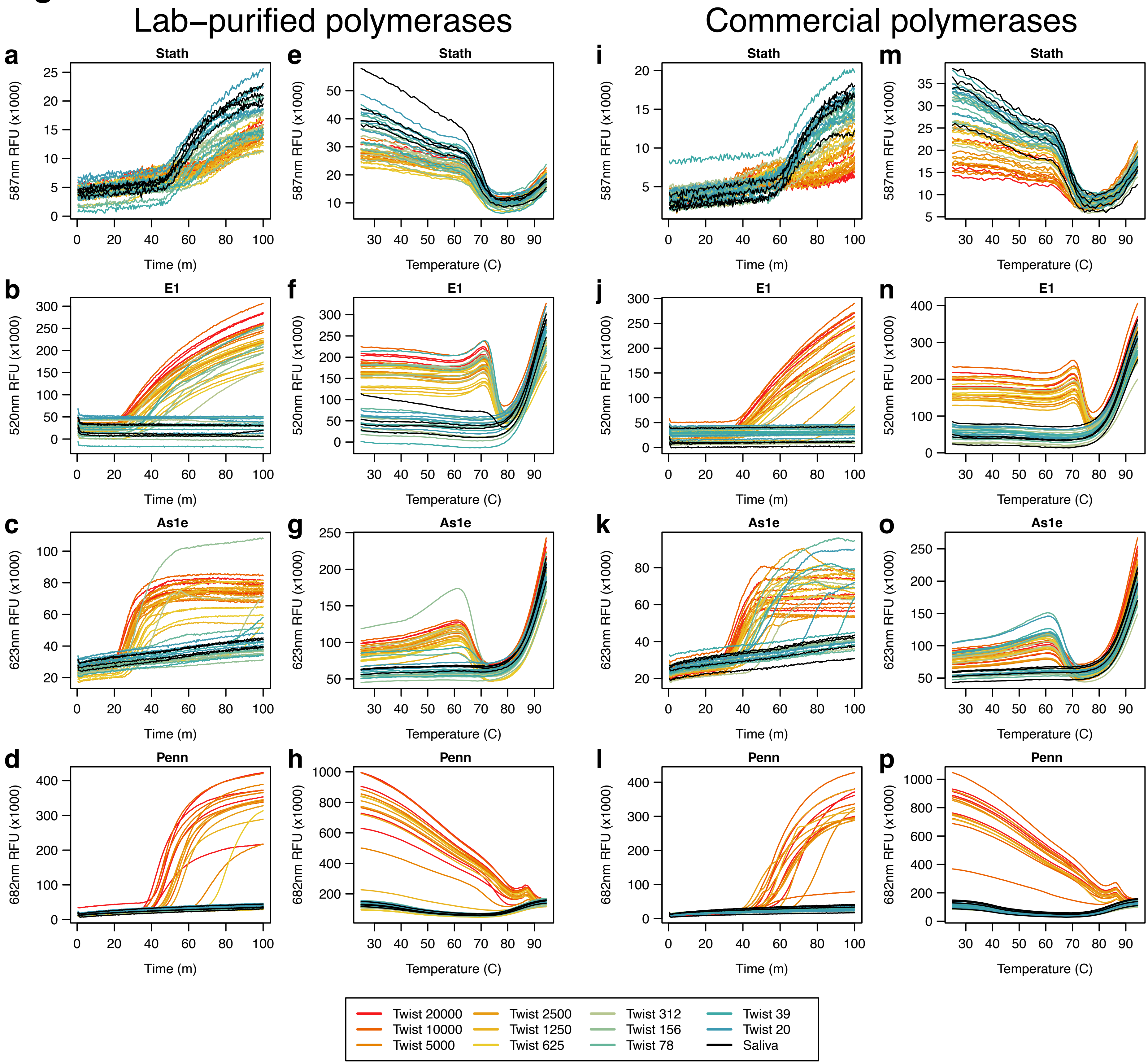

Figure S3. Comparison of laboratory-purified and commercial polymerase enzymes using a quadruplex LAMP-BEAC assay of saliva samples spiked with synthetic SARS-CoV-2 RNA. Assays were carried out using an amplicon to detect human STATH RNA (A) and three amplicons to detect SARS-CoV-2 (B-D). For these assays, synthetic SARS-CoV-2 RNA was diluted into saliva (inactivated as described[9]); copies per microliter are shown by the color code in the lower right. For A-D, the x-axis shows time after starting the assay, and the y-axis shows fluorescence intensity. E-H shows melt curve analysis for samples in A-D. For E-H, the x-axis shows temperature, and the y-axis shows fluorescence intensity. (I-P) Assays are exactly as in A-H, but commercial reverse transcriptase and DNA polymerase (NEB Warm Start LAMP Kit master mix product number E1700L) were used instead of the locally designed and purified polymerases.
