# Supplementary material for: Detection of SARS-CoV-2 RNA using RT-LAMP and molecular beacons
Source: Genome Biol. 2021 Jun 3;22:169. doi: 10.1186/s13059-021-02387-y (PMC8173101; doi:10.1186/s13059-021-02387-y)
Supplement: Supplementary file 4 — Additional file 4. Review history. [file 13059_2021_2387_MOESM4_ESM.docx]

**1^st^ round**

**Reviewer 1**

"LAMP-BEAC: Detection of SARS-CoV-2 RNA Using RT-LAMP and Molecular Beacons" by Dr Frederic Bushman and colleagues describes the development of a novel method for detecting SARS-CoV-2, which is designed to overcome supply chain bottlenecks, be amenable to use in low resource settings, and to provide rapid results. While several other groups have used LAMP to detect SARS-CoV-2, this study incorporates molecular beacon probes to substantially improve background and to reduce spurious positive signals often seen with colorimetric or fluorescent detection. The authors optimize the molecular beacon to perform well at the temperatures required for isothermal amplification in a LAMP assay and develop multiplexed LAMP assays for multiple viral targets as well as a human control to assess the success of sample collection. The authors develop a protocol based on saliva samples and benchmark these assays using both synthetic RNA and patient samples (including NP swabs) by comparing to RT-qPCR results. Finally, the authors report the development of protocols to purify enzymes in order to reduce costs and to mitigate potential supply chain disruption.

In general, I think that the incorporation and optimization of the molecular beacon probe into the LAMP assay makes a substantial improvement and this paper describes an assay that is potentially useful for other researchers (with the caveat that assay sensitivity is reduced compared to RT-qPCR assays). There are a few issues with clarity and completeness of descriptions in the text that should be addressed and the authors should also do more to describe and substantiate the use-cases for this assay (see below). However, assuming that these minor issues are addressed I support publication of this manuscript in Genome Biology.

Specific comments:

-Figure 1 is not referenced in the portion of the text describing the assay (not mentioned until after Figure 2) - this figure could also be improved to aid in interpreting the subsequent figures by marking the locations of the different targets analyzed (and labeling the associated genes) on the SARS-CoV-2 RNA genome.

Figure 2 - The authors should specify the viral target sequence in the text and figure or legend. For consistency, I would also suggest keeping the same (left-right) relative orientation of the amplification and melt curve panels in Figures 2 and 3.

Figure 3 - I can't see the negative control in these plots. I would assume it is hidden under some of the other non-amplifying lines (if this is not the case, I would need to re-evaluate this data), but since this data is critical to interpreting this figure, the authors should make sure that the negative control is visible in the figure panels.

-It does not really look like the signal intensity or timing tracks with viral copy number. Given this, it would be useful to see some data on reproducibility. It looks like there may be replicates in these plots. Are these technical replicates within a run or independent experiments. Some measure of assay variance/reproducibility between fully independent experiments would be useful here.

-Figure 4 -Did the 3 clinical positive samples which did not amplify for the human target have high Ct values for the human control in the RT-qPCR assay?

-The section of the text describing Figure 4 is written in a confusing manner. With close reading, one can infer that the 22 selected clinical samples were NP positives based on the clinical diagnostic assay, but it would be nice to have this spelled out more clearly. Also, since there is a complication of several different sample types (NP and saliva) as well as multiple saliva replicates per patient, it would be useful to have a schematic outlining this experimental design and highlighting which samples and data were used in the Figure. The authors could include sensitivity and specificity numbers in the text both for all samples and for the subset of samples with >1,000 copies/ul by RT-qPCR.

-Another thing that should be clarified in this section is whether the additional 56 saliva samples tested were negative patient samples, or if these were randomly selected (and a mix of positives and negatives)?

-The purification of custom enzymes is described at a high level in the methods. To make this information useful for the community, a detailed protocol should be provided either in the methods, as a supplement, or on an external protocol site. Additionally, expression constructs should be made available through Addgene or another public repository.

-It would be useful for the authors to comment on the instrumentation required to carry out these multiplexed endpoint measurements and their cost compared to the qPCR machines used in this study. This is important for assessing the benefit of this assay for application in low resource settings or even point-of-care.

-In discussing SalivaDirect, the authors should point out other important differences, such as the fact that SalivaDirect is an FDA EUA cleared assay (and thus has undergone a more rigorous clinical validation) and is more sensitive than the LAMP-BEAC assay. It is also difficult to imagine the LAMP-BEAC assay being used for routine clinical diagnostics given the sensitivity, so I think that a more compelling point to make in the discussion would be to highlight the potential utility of massive-scale assurance testing in healthy populations to screen for asymptomatic carries with relatively high viral loads and/or for use in low-resource settings. Finally, RT-qPCR is not terribly expensive on a per assay basis, so it would be useful to have some sort of cost comparison between LAMP-BEAC (+/- homemade enzymes) and the RT-qPCR CDC assay. The utility in low resource or especially high-throughput settings will depend critically on these economics. It might also be useful/interesting to cite some of the modeling literature here that makes the case of radically larger-scale testing (particularly as it relates to how even a test with lower sensitivity can be useful if applied at a population scale).

**Reviewer 2**

Colorimetric LAMP is a popular choice for SARS-CoV-2 diagnostics as it gives a quick color readout. But, this method has a false positive problem and does not allow multiplexing. Several attempts have been made by the field to attempt to address these limitations. Sherrill-Mix et al., continue this trend by developing a SARS-CoV-2 LAMP-BEAC method that pairs LAMP with molecular beacons. Their method combines together a number of assay features that other have used to develop an iNAAT that can discriminate specific and non-specific amplification and also allow multiplexing of targets. They show proof of concept experiments and apply the assay to COVID-19 clinical samples. While the paper does make some steps forward, I find it currently a bit limited in what it delivers. There is definitely a need for better COVID assays, whether the assay will make a difference really comes down to sensitivity and selectivity. In order to really have an impact I believe the manuscript still has additional work.

Major comment:

The central result of the paper is that they have developed a more specific multiplexable version of LAMP to allow for SARS-CoV-2 detection. The authors show several tests of performance and should be commended for their clinical samples but the performance on these samples didn't appear to be that great. Overall, I found it quite difficult to assess performance of their assay compared to other lamp assays. For example, if I read their paper correctly their sensitivity is comparable or worse to some of the other reported LAMP assay. A limit of detection assay such as the type required for EUA submission would be extremely helpfully in bench marking this assay versus other assays and maybe a side-by-side of their LAMP-BEAC versus other LAMP assays on the same clinical samples (or minimally contrived samples). There have been a lot of SARS-CoV-2 LAMP assays developed and if this assay doesn't offer a better LoD, it is going to be hard for it to gain traction.

Alternatively, the authors might be able to garner more interest by better characterizing the beacon design for others in the scientific community who are interested to adapt it. It seems that the authors merely designed 4 beacons on 4 different LAMP targets based on the published LAMP primer availability. It would be resourceful to other scientists who want to apply this method to other targets to know where to insert the lock nucleic acids into the beacon design and how many to insert, for example. What is the G/C content that one should be aiming for when designing these beacons? Additionally, how did you determine the optimal beacon concentration to use for LAMP-BEAC? The authors have not addressed the beacon design, which is the main difference in this study, with enough experiments or rigor.

Minor points:

Abstract: dial back the claim: "assay suitable for population screening."

Fig 2. Add replicate (biological or technical replicates?) information for the experiments.

Fig 2a and b. Add specific time values of when fluorescent signals come up into the manuscript text. It seems LAMP-BEAC is slower than normal LAMP, one but cannot tell without values.

Fig 3a. Where is the negative control curve for STATH-LB without saliva?

Fig 3d. Address why Penn-LF beac assay performs 10-fold worse in contrived saliva compared to Fig 2 experiments? Is it due to multiplexing or contrived samples?

Fig S2. The figures of lab-purified polymerase and commercial polymerases side-by-side assays are identical. Need to fix.

**Authors’ response**

Reviewer 1.

We are pleased that the reviewer felt “In general, I think that the incorporation and optimization of the molecular beacon probe into the LAMP assay makes a substantial improvement and this paper describes an assay that is potentially useful for other researchers”.

Specific comments:

Reviewer’s comment: -Figure 1 is not referenced in the portion of the text describing the assay (not mentioned until after Figure 2) - this figure could also be improved to aid in interpreting the subsequent figures by marking the locations of the different targets analyzed (and labeling the associated genes) on the SARS-CoV-2 RNA genome.

Response: Thank you for the useful suggestion. We have added the SARS-CoV-2 genetic map as Fig. 1c as suggested, and add an additional call out to the figure.

Reviewer’s comment: Figure 2 - The authors should specify the viral target sequence in the text and figure or legend. For consistency, I would also suggest keeping the same (left-right) relative orientation of the amplification and melt curve panels in Figures 2 and 3.

Response: Again thank you for the helpful suggestion—we now specify the target sequence.

Reviewer’s comment: Figure 3 - I can't see the negative control in these plots. I would assume it is hidden under some of the other non-amplifying lines (if this is not the case, I would need to re-evaluate this data), but since this data is critical to interpreting this figure, the authors should make sure that the negative control is visible in the figure panels. Response: We have adjusted the plot to make the negative controls more obvious. Reviewer’s comment: -It does not really look like the signal intensity or timing tracks with viral copy number. Given this, it would be useful to see some data on reproducibility. It looks like there may be replicates in these plots. Are these technical replicates within a run or independent experiments. Some measure of assay variance/reproducibility between fully independent experiments would be useful here.

Response: We have added Figure 5 showing data from 480 reactions to addresses these points.

Reviewer’s comment: -Figure 4 -Did the 3 clinical positive samples which did not amplify for the human target have high Ct values for the human control in the RT-qPCR assay?

Response: We now address this point in the text. The samples were detectable by CDC’s RNaseP primers.

Reviewer’s comment: -The section of the text describing Figure 4 is written in a confusing manner. With close reading, one can infer that the 22 selected clinical samples were NP positives based on the clinical diagnostic assay, but it would be nice to have this spelled out more clearly. Also, since there is a complication of several different sample types (NP and saliva) as well as multiple saliva replicates per patient, it would be useful to have a schematic outlining this experimental design and highlighting which samples and data were used in the Figure. The authors could include sensitivity and specificity numbers in the text both for all samples and for the subset of samples with >1,000 copies/ul by RT-qPCR.

Response: We have repeated the assay on additional samples, edited the text to increase clarity, and added discussion on sensitivity and specificity for these samples. To show assay robustness and sensitivity in more detail, we have added the extensive new analysis presented in Figure 5.

Reviewer’s comment: -Another thing that should be clarified in this section is whether the additional 56 saliva samples tested were negative patient samples, or if these were randomly selected (and a mix of positives and negatives)?

Response: The samples were a mixture of positives and negatives. We have rewritten the indicated section to clarify this.

Reviewer’s comment: -The purification of custom enzymes is described at a high level in the methods. To make this information useful for the community, a detailed protocol should be provided either in the methods, as a supplement, or on an external protocol site. Additionally, expression constructs should be made available through Addgene or another public repository.

Response: We have developed the protocol further for clarity. We are in the process of depositing the expression constructs at Addgene.

Reviewer’s comment: -It would be useful for the authors to comment on the instrumentation required to carry out these multiplexed endpoint measurements and their cost compared to the qPCR machines used in this study. This is important for assessing the benefit of this assay for application in low resource settings or even point-of-care.

Response: We have added a panel to Figure 1 showing visual detection of these assays. We now point out that with visual endpoint detection, all that is required is a heatblock/incubator and cheap fluorescent viewer. We also have added more details on the instruments used for analysis.

Reviewer’s comment: -In discussing SalivaDirect, the authors should point out other important differences, such as the fact that SalivaDirect is an FDA EUA cleared assay (and thus has undergone a more rigorous clinical validation) and is more sensitive than the LAMP-BEAC assay. It is also difficult to imagine the LAMP-BEAC assay being used for routine clinical diagnostics given the sensitivity, so I think that a more compelling point to make in the discussion would be to highlight the potential utility of massive-scale assurance testing in healthy populations to screen for asymptomatic carries with relatively high viral loads and/or for use in low-resource settings. Finally, RT-qPCR is not terribly expensive on a per assay basis, so it would be useful to have some sort of cost comparison between LAMP-BEAC (+/- homemade enzymes) and the RT-qPCR CDC assay. The utility in low resource or especially high-throughput settings will depend critically on these economics. It might also be useful/interesting to cite some of the modeling literature here that makes the case of radically larger-scale testing (particularly as it relates to how even a test with lower sensitivity can be useful if applied at a population scale).

Response: We have added an entirely new section, showing that increasing the assay volume allows much lower detection of RNA copies per unit volume. This does not work with conventional detection. We have added a breakdown of the costs for molecular beacons (pennies per reaction when ordered from commercial vendors).

Reviewer 2.

We are gratified that the reviewer felt “the paper does make some steps forward”, but we recognize that the reviewer wanted more evidence of impact.

Response: Our best evidence for impact, as mentioned above, is that the LAMP-BEAC assay is now CLIA approved, and in use to screen thousands of asymptomatic subjects each week at the University of Pennsylvania School of Medicine (more than 45,000 tests). In the course of this, more than 40 asymptomatic but SARS-CoV-2- infected people have been identified and engaged in protocols to prevent further spread. We believe that this has protected large numbers of people from infection, which we believe is the clearest impact of this body of work.

Reviewer’s comment: The central result of the paper is that they have developed a more specific multiplexable version of LAMP to allow for SARS-CoV-2 detection. The authors show several tests of performance and should be commended for their clinical samples but the performance on these samples didn't appear to be that great. Overall, I found it quite difficult to assess performance of their assay compared to other lamp assays. For example, if I read their paper correctly their sensitivity is comparable or worse to some of the other reported LAMP assay. A limit of detection assay such as the type required for EUA submission would be extremely helpfully in bench marking this assay versus other assays and maybe a side-by-side of their LAMP-BEAC versus other LAMP assays on the same clinical samples (or minimally contrived samples). There have been a lot of SARS-CoV-2 LAMP assays developed and if this assay doesn't offer a better LoD, it is going to be hard for it to gain traction.

Response: In response to the reviewer’s comment, we have added more panels comparing LAMP-BEAC to conventional LAMP. Note that each of the LAMP primer sets used is from a previous report—our contribution it to tighten up the specificity with detection by molecular beacons. Regarding regulatory approval, as mentioned, the assay has been CLIA approved and used to screen 45,000 samples.We have added an additional section and figure (Fig. 5) comparing standard fluorometric LAMP against LAMP-BEAC and show that LAMP-BEAC can greatly improve sensitivity.

Reviewer’s comment: Alternatively, the authors might be able to garner more interest by better characterizing the beacon design for others in the scientific community who are interested to adapt it. It seems that the authors merely designed 4 beacons on 4 different LAMP targets based on the published LAMP primer availability. It would be resourceful to other scientists who want to apply this method to other targets to know where to insert the lock nucleic acids into the beacon design and how many to insert, for example. What is the G/C content that one should be aiming for when designing these beacons? Additionally, how did you determine the optimal beacon concentration to use for LAMP-BEAC? The authors have not addressed the beacon design, which is the main difference in this study, with enough experiments or rigor.

Response: We have added further detail to how we designed our beacons. In addition, we now show all iterations of beacons made and tested in this study (Table S1 and Figure S3). We hope this will aid others in the design of further beacons.

Minor points:

Reviewer’s comment: -Abstract: dial back the claim: "assay suitable for population screening."

Response: In the paper, we now specify how the assay has been used for population screening (45,000 tests run; >40 positive subjects referred to follow up).

Reviewer’s comment: -Fig 2. Add replicate (biological or technical replicates?) information for the experiments.

Response: We have added the data from an additional 480 reactions in new Figure 5.

Reviewer’s comment: -Fig 2a and b. Add specific time values of when fluorescent signals come up into the manuscript text. It seems LAMP-BEAC is slower than normal LAMP, one but cannot tell without values.

Response: We have now extensively tested time to threshold between fluorometric LAMP and LAMP-BEAC in new Figure 5.

Reviewer’s comment: -Fig 3a. Where is the negative control curve for STATH-LB without saliva?

Response: Such negative controls are shown in Figure S3.

Reviewer’s comment: -Fig 3d. Address why Penn-LF beac assay performs 10-fold worse in contrived saliva compared to Fig 2 experiments? Is it due to multiplexing or contrived samples?

Response: We now explain that competition between amplicons results in lower performance for Penn in multiplex settings.

Reviewer’s comment: -Fig S2. The figures of lab-purified polymerase and commercial polymerases side-by-side assays are identical. Need to fix.

Response: We noticed this after submitting the paper and sent a revised figure to Genome Biology. Apparently the corrected figure did not make it out to reviewers. We have corrected the figure in the revised draft.

**2^nd^ round**

**Reviewer 1**

The revision for "LAMP-BEAC: Detection of SARS-CoV-2 RNA Using RT-LAMP and Molecular Beacons" by Dr Frederic Bushman and colleagues addressed the primary concerns of the reviewers and demonstrated that substantially improved sensitivity could be achieved by simply scaling up the reaction volume. I support publication of this manuscript in Genome Biology. A couple of small comments that could improve the clarity of the final version are below:

1) Abstract line 15-16 - I would not characterize RT-qPCR tests as expensive. It's true that many providers are charging $80-100 for these tests, but they can be run quite inexpensively.

2) Background line 29 - SARS-CoV-2 was detected before March 2020 (The unusual pneumonia cluster was detected in December 2019 and the virus was identified in January 2020). The numbers of deaths and cases in this sentence are already sadly out of date and should be updated.

3) Discussion - page 23, lines 41-43 - based on my understanding of CLIA, I am not sure if it is appropriate to say that this is a CLIA "approved" LDT, since unlike FDA EUA this does not necessarily mean that an external regulator has signed off on the test, rather that the developing lab and their medical director feel comfortable with the validation state of the assay. I would rephrase to say that the assay is currently being run as a CLIA LDT (as opposed to saying the assay has received approval, unless the lab has indeed been recently inspected and the assay has been explicitly "approved" in some manner).

**Reviewer 2**

The additional information, figures, and textual clarifications have improved the paper. I would still point out that EUA approval and CLIA approval are a world of difference. CLIA approval is more about if your lab is running with good practices and does your medical director sign off on the assay. EUA approval is more about the assay than the lab. It is unclear to me whether this assay would get EUA approval. It probably would, but not 100% clear.

Also, while it is clear the assay is catching positive cases, the question is how many cases are missed compared to an alternative assay. Another way of asking this is how many cases of person-to-person transmission are there at U Penn and how does this compare with the background transmission rate in the community and to other settings using asymptomatic surveillance.

With this all said, I do believe this types of home brewed university testing programs have been super important and are worth being published. It will be useful in the aftermath of COVID-19 to analyze all these programs and determine what was effective and if regulations needed to be changed to facilitate these type of tests in the future.

**Authors’ response**

**Reviewer 1**

1)Abstract line 15-16 - I would not characterize RT-qPCR tests as expensive. It's true that many providers are charging $80-100 for these tests, but they can be run quite inexpensively.

- We have rephrased the indicated section as “instrument costs are high”.

2)Background line 29 - SARS-CoV-2 was detected before March 2020 (The unusual pneumonia cluster was detected in December 2019 and the virus was identified in January 2020). The numbers of deaths and cases in this sentence are already sadly out of date and should be updated.

-We have updated as suggested.

3)Discussion - page 23, lines 41-43 - based on my understanding of CLIA, I am not sure if it is appropriate to say that this is a CLIA "approved" LDT, since unlike FDA EUA this does not necessarily mean that an external regulator has signed off on the test, rather that the developing lab and their medical director feel comfortable with the validation state of the assay. I would rephrase to say that the assay is currently being run as a CLIA LDT (as opposed to saying the assay has received approval, unless the lab has indeed been recently inspected and the assay has been explicitly "approved" in some manner).

-We have rephrased this section to “has been implemented in a CLIA certified laboratory” as suggested.

**Reviewer 2**

The additional information, figures, and textual clarifications have improved the paper. I would still point out that EUA approval and CLIA approval are a world of difference. CLIA approval is more about if your lab is running with good practices and does your medical director sign off on the assay. EUA approval is more about the assay than the lab. It is unclear to me whether this assay would get EUA approval. It probably would, but not 100% clear.

-The reviewer makes a good point; we have rephrased this section.

Also, while it is clear the assay is catching positive cases, the question is how many cases are missed compared to an alternative assay. Another way of asking this is how many cases of person-to-person transmission are there at U Penn and how does this compare with the background transmission rate in the community and to other settings using asymptomatic surveillance.

-We do not know of any cases of transmission of SARS-CoV-2 within the UPenn medical school population tested by the screening program. Another manuscript is being written describing the logistics and outcome of the Penn screening program.

With this all said, I do believe this types of home brewed university testing programs have been super important and are worth being published. It will be useful in the aftermath of COVID-19 to analyze all these programs and determine what was effective and if regulations needed to be changed to facilitate these type of tests in the future.

-We strongly agree, and very much hope that the US and the world can be better prepared for the next epidemic.
